# Supplementary figures and images for: Modeling human B cell development with pluripotent stem cells
Source: bioRxiv. 2026 May 7:2026.05.04.722674. Preprint. [Version 1] doi: 10.64898/2026.05.04.722674 (PMC13174478; doi:10.64898/2026.05.04.722674)

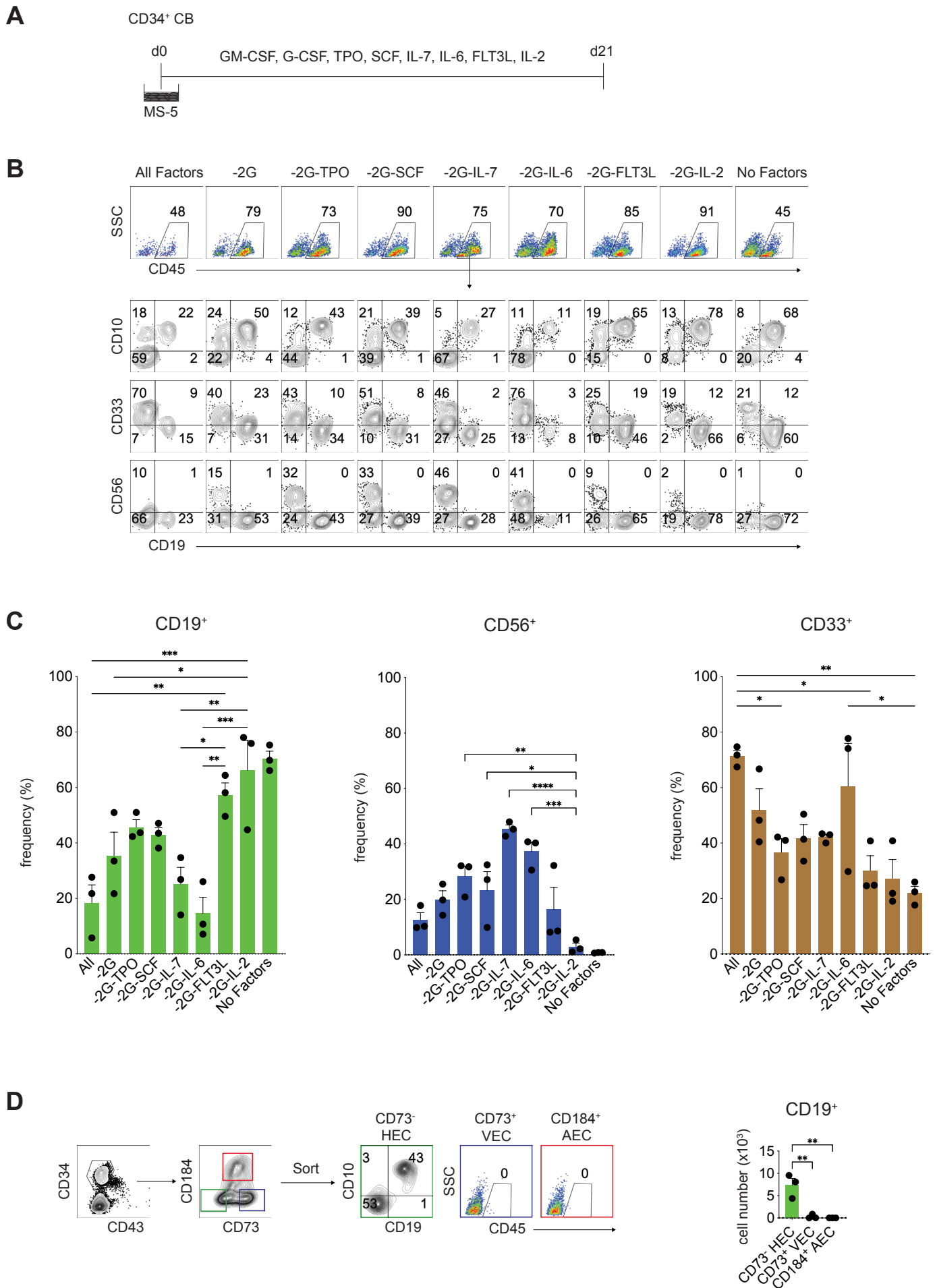

**A**

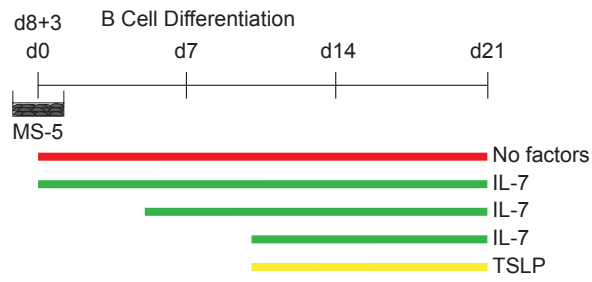

**B**

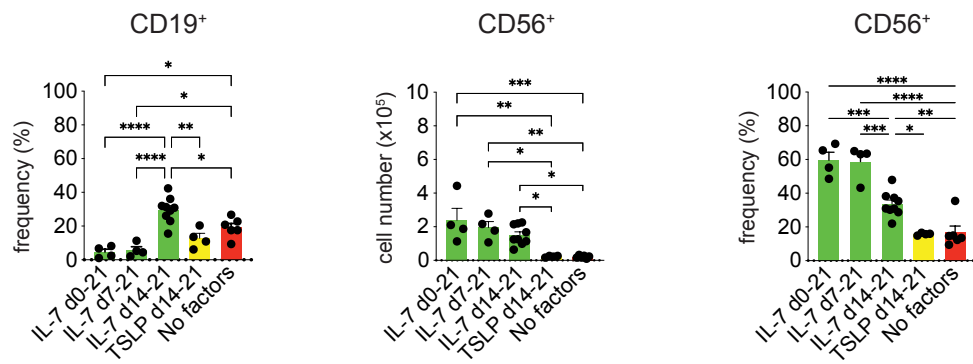

**C**

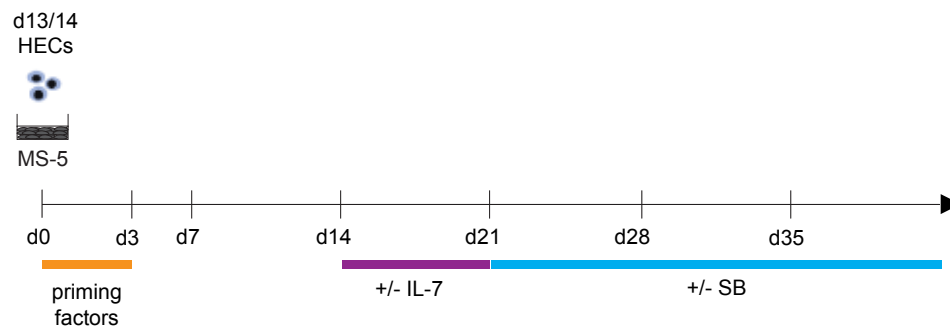

**A** Expression level of genes from Large pre-B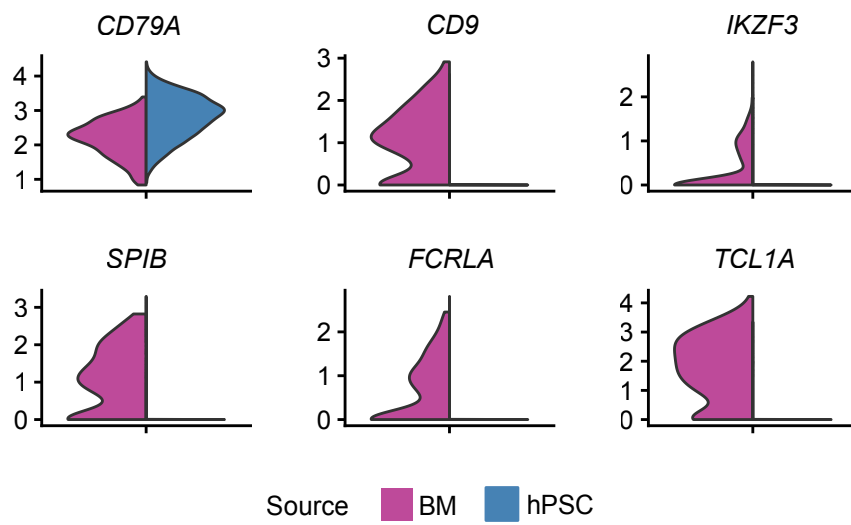

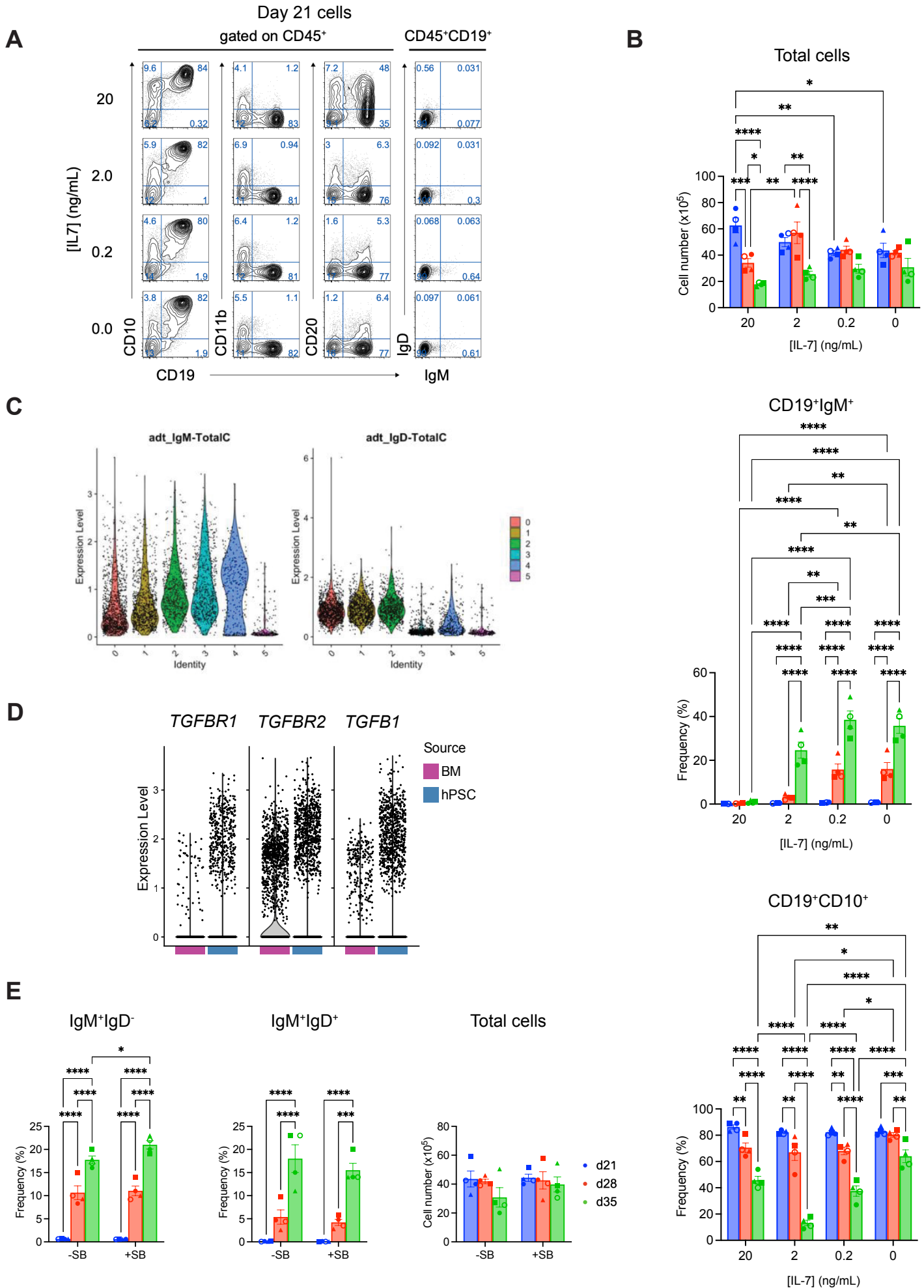

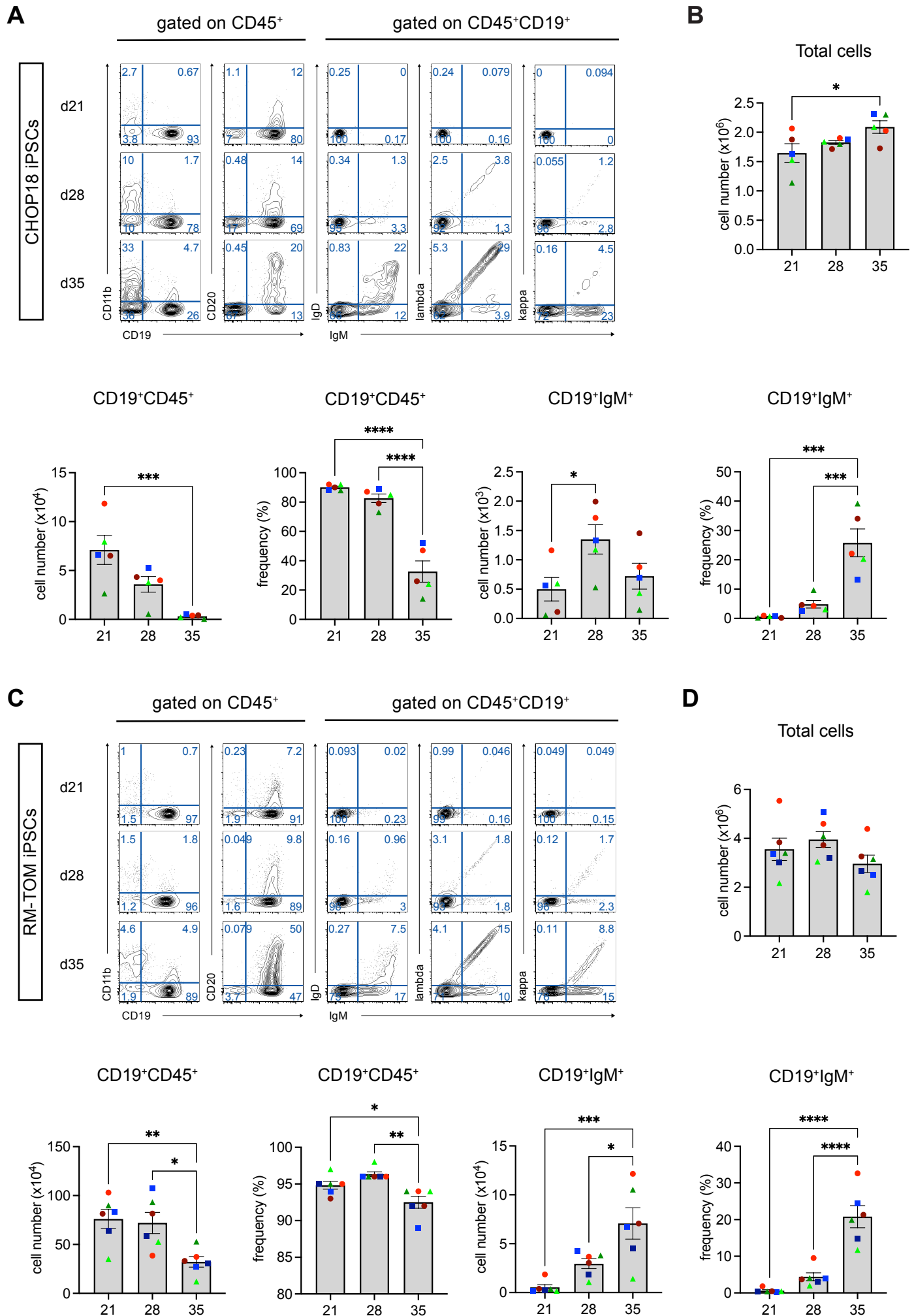

Supplementary Figure 6

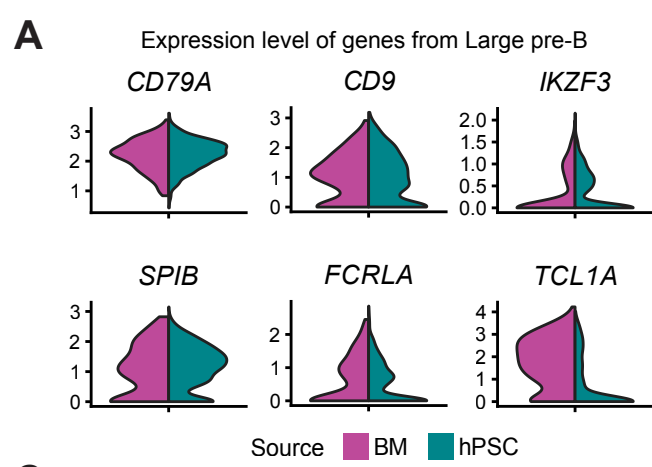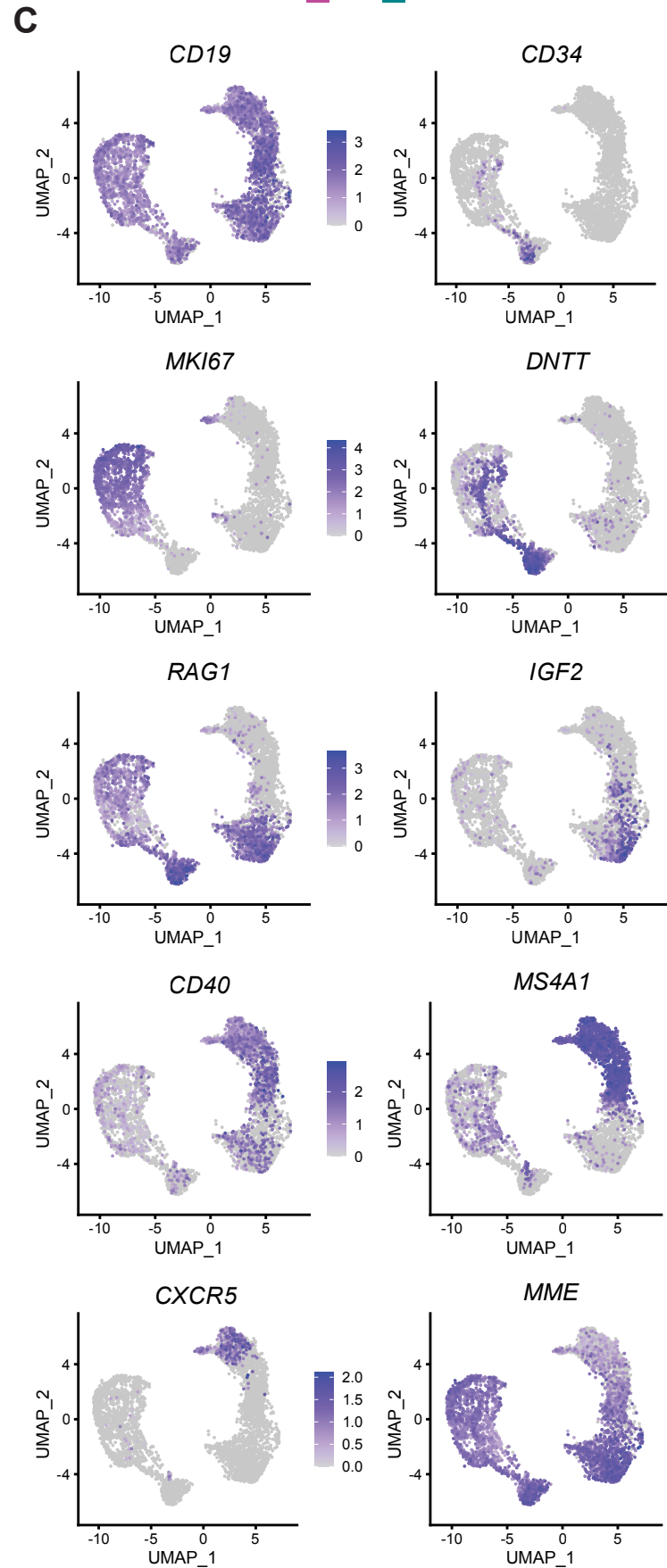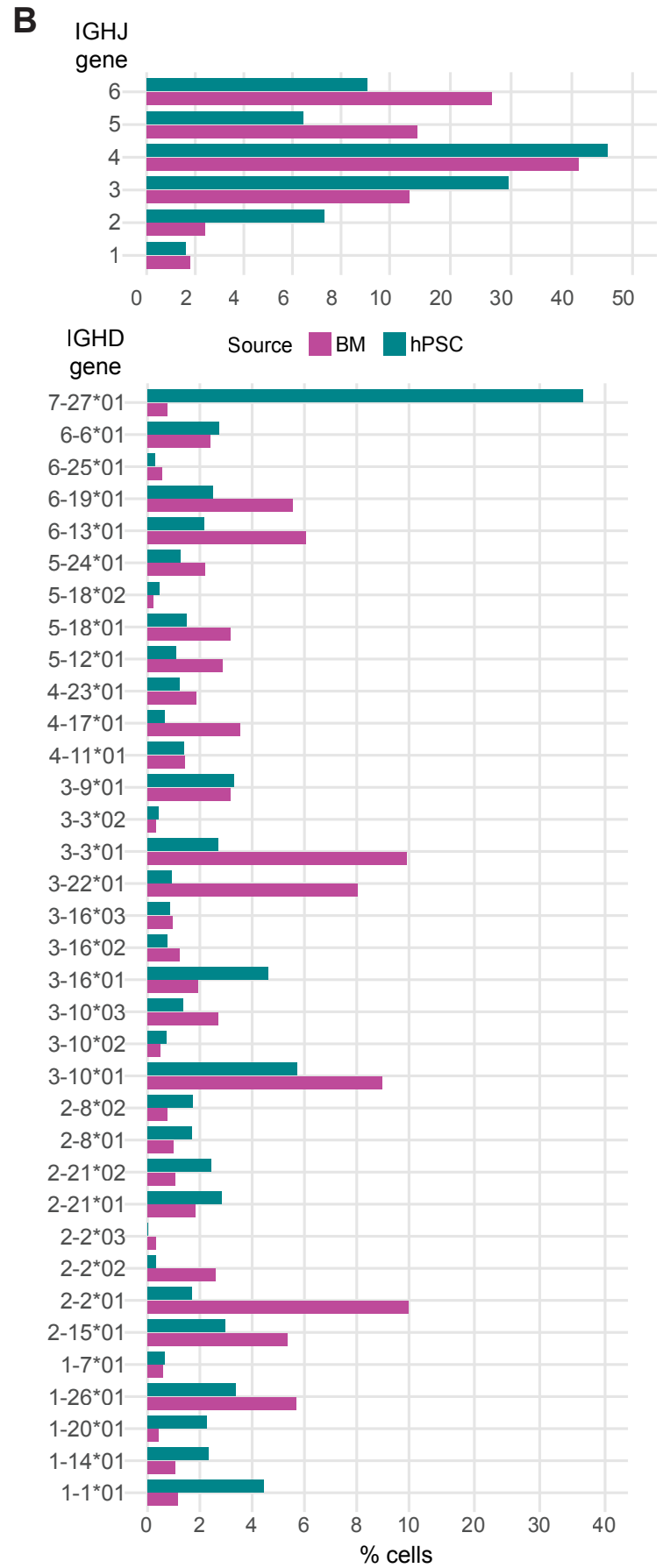

**A**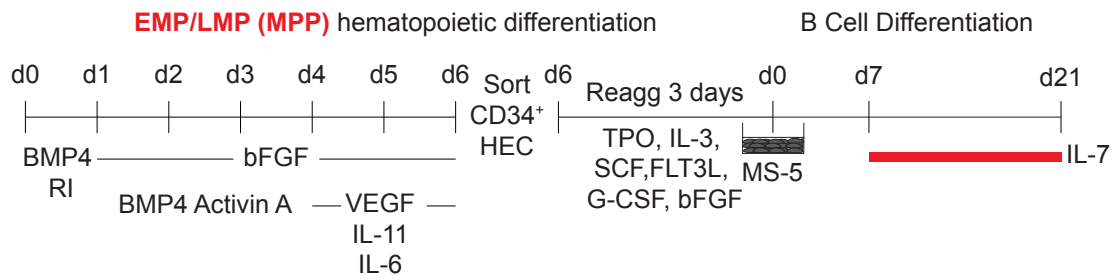**B**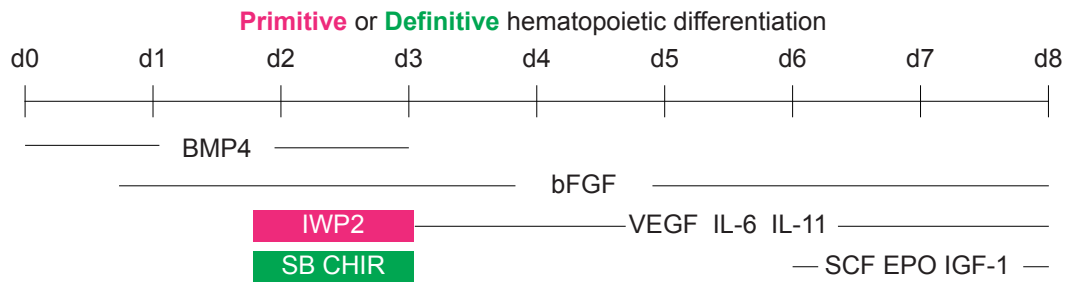**C**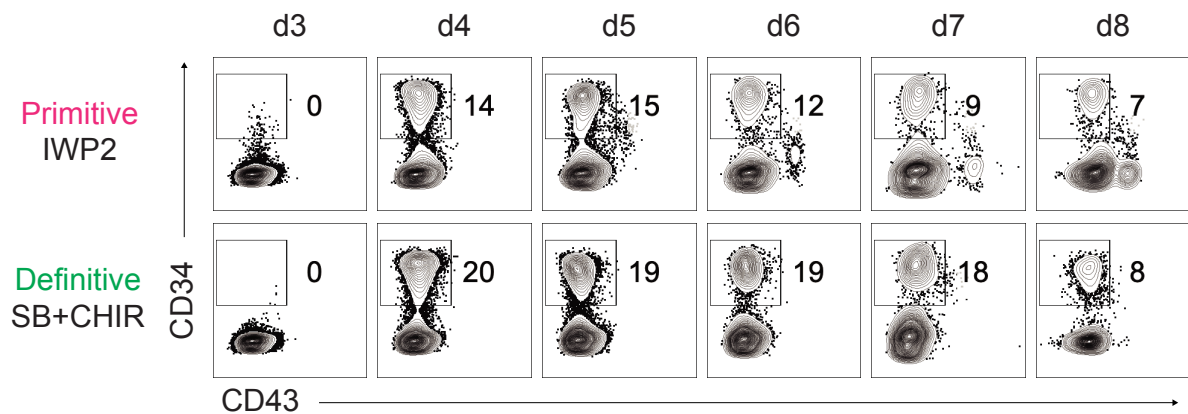**D**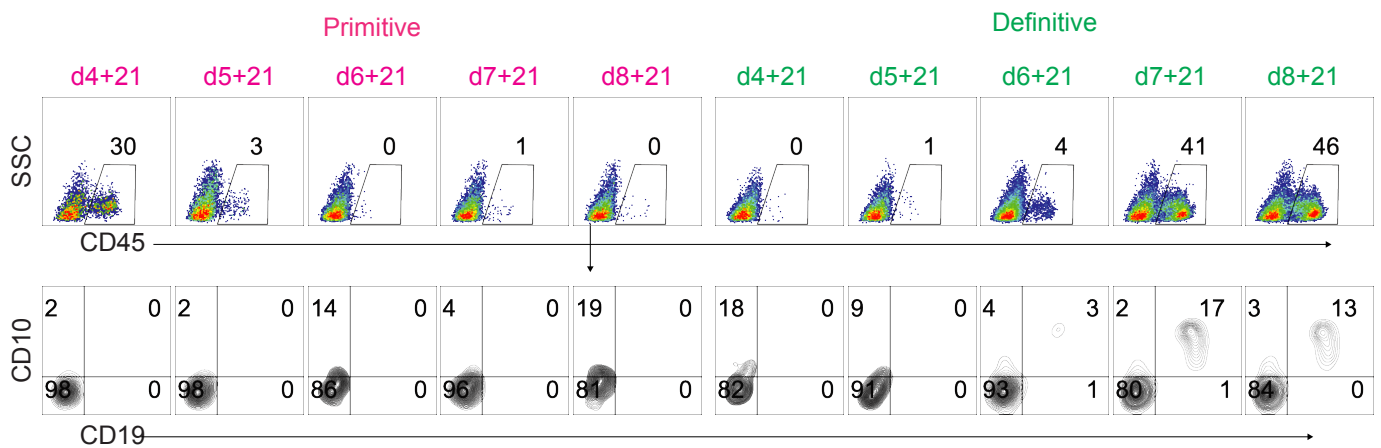

Supplement: Supplement 1 — Supplementary Figure 1. B cell development from cord blood- and hPSC-derived progenitors (A) Scheme of protocol used to generate B cells from CD34+ cord blood (CB) progenitors. (B) Representative flow cytometric analysis of CB-derived populations generated in the different cytokine combinations following 21 days of MS-5 co-culture. The CD19, CD10, CD33, CD56 profiles are from gated CD45+DAPI− cells. 2G: GM-CSF, G-CSF. Cells cultured in the absence of factor represent the negative control. (C) Quantification of the frequency of CD19+ B cells, CD56+ NK cells and CD33+ myeloid cells generated in each of the indicated cytokine combinations n ≥ 3. (D) Left: Representative flow cytometric analyses of expression of indicated markers on day 8 CD34+ fractions [CD184−CD73−, HECs; CD184+CD73lo, arterial endothelial cells (AEC); CD184−CD73+, venous endothelial cells (VECs)] and on the populations generated from them following 21 days of culture on MS-5 stroma (right). The indicated populations were isolated by FACS, the cells aggregated with hematopoietic factors for 2 days and the aggregates cultured in the absence of factors (No factors). Right: Quantification of the number of CD19+ B cells generated from day 8 HECs, VECs, and AECs. n = 3. Error bars represent SEM. *P < 0.05, **P < 0.01, ***P < 0.001, ****P < 0.0001, ##P < 0.01, ###P < 0.001, ####P < 0.0001 by one way ANOVA analyses with Tukey’s multiple comparisons. Supplementary Figure 2. Effect of IL-7 on B cell development from hPSC-derived progenitors (A) Scheme of the protocol used to test the effect of IL-7 or TSLP on the generation of CD19+ cells from day 8 HECs. The factors were added during the indicated time intervals. (B) Quantification of the frequency of CD19+ B cells and of the total number and frequency of CD56+ NK cells generated from day 8 hPSC-derived HEC cultured for 21 days under the indicated conditions. Error bars represent SEM. *P < 0.05, **P < 0.01, ***P < 0.001, ****P < 0.0001, #P < 0.05 by one way A [file media-1.pdf]
